# Assembling GHERG: Could “academic crowd–sourcing” address gaps in global health estimates?

**DOI:** 10.7189/jogh.05.010101

**Published:** 2015-06

**Authors:** Igor Rudan, Harry Campbell, Ana Marušić, Devi Sridhar, Harish Nair, Davies Adeloye, Evropi Theodoratou, Kit Yee Chan

**Affiliations:** 1 Centre for Global Health Research, The Usher Institute for Population Health Sciences and Informatics, University of Edinburgh, Scotland, UK; 2 Croatian Centre for Global Health, University of Split School of Medicine, Split, Croatia; 3 Department of Research in Biomedicine and Health, University of Split School of Medicine, Split, Croatia

## Abstract

In recent months, the World Health Organization (WHO), independent academic researchers, the Lancet and PLoS Medicine journals worked together to improve reporting of population health estimates. The new guidelines for accurate and transparent health estimates reporting (likely to be named GATHER), which are eagerly awaited, represent a helpful move that should benefit the field of global health metrics. Building on this progress and drawing from a tradition of Child Health Epidemiology Reference Group (CHERG)’s successful work model, we would like to propose a new initiative – “Global Health Epidemiology Reference Group” (GHERG). We see GHERG as an informal and entirely voluntary international collaboration of academic groups who are willing to contribute to improving disease burden estimates and respect the principles of the new guidelines – a form of “academic crowd–sourcing”. The main focus of GHERG will be to identify the “gap areas” where not much information is available and/or where there is a lot of uncertainty present about the accuracy of the existing estimates. This approach should serve to complement the existing WHO and IHME estimates and to represent added value to both efforts.

Rapid development of information technologies in the past two decades has brought about major improvements in the generation, sharing and analysis of information on the health status of the entire human population. This has facilitated the development of the field of “Global Health Metrics”, which has its champions in World Health Organization's Mortality and Burden of Disease Unit and University of Washington’s Institute for Health Metrics and Evaluation (IHME). A number of other groups, initiatives and institutions also contribute substantially to this field and regularly publish population health estimates. These include national epidemiological services, population–specific (eg, occupational, ethnic, age–and gender–defined) and disease–specific registries, academic research groups interested in specific health problems, professional societies that specialize in a particular disease(s), and initiatives by international organizations.

Examples of organizations specializing in population health surveillance that require health metrics as a starting point include the United States Centers for Disease Control and Prevention (USCDCP), European Centre for Disease Prevention and Control (ECDPC), Chinese Center for Disease Control and Prevention (CCDCP), International Agency for Research on Cancer (IARC) and the United Nation’s Children Fund (UNICEF). All of these organizations regularly publish summaries of large amounts of information on population health that is collected through their services. Moreover, the Demographic and Health Surveys (DHS) Program, supported by USAID, continues to collect, analyze and disseminate representative data on population, health, HIV, and nutrition through more than 300 nationally–representative household surveys in over 90 countries. Similarly, UNICEF assists countries in collecting and analyzing data on health of women and children through its household survey program called Multiple Indicator Cluster Surveys (MICS).

Further to these efforts, professional societies such as the International Diabetes Federation (IDF) and Alzheimer Disease International (ADI) work together with the World Health Organization to develop and regularly update the global, regional, and sometimes national estimates of type 2 diabetes and dementia, respectively. There are many other examples of successful collaborations between international agencies and academic research groups in order to develop accurate and transparent population health estimates. Some of the most notable examples are the United Nation’s Inter–Agency Group for Child Mortality Estimation (IGME), and the Child Health Epidemiology Reference Group (CHERG), in which our group in Edinburgh has also been involved for the past decade. IGME regularly revises the overall child mortality estimates at the national level, while CHERG made major contributions to the understanding of the leading causes of child deaths globally, regionally and nationally. CHERG’s work provided much needed evidence to inform and help direct policies towards achieving UN’s Millennium Development Goal 4. CHERG developed into a collaboration between the WHO, UNICEF and a group of independent technical experts from leading academic institutions who worked together to assemble available information on the causes of child deaths in parts of the world where there was no adequate vital registration coverage. An important aspect of CHERG work was to critically review this information and to include only data which met stated quality criteria regarding validity and representativeness.

In addition to understanding the burden of disease in human populations, determinants of the burden – ie, the underlying risk factor causes that make people ill – are also of interest to the field of global health metrics. Several countries or regions have set up large–scale biobanks in recent years to study determinants of population health on a very large scale, using a “big data” approach. Some of the most impressive examples are UK Biobank, the Kadoorie biobank in China, and the EPIC prospective study in Europe. Each one of these biobanks includes about 500 000 persons which should give them sufficient study power to tease out the effects of many different potential environmental, genetic and lifestyle contributors to human diseases. Likewise, there are academic groups that regularly review and assess the scientific literature to identify risk factors through large meta–analyses, such as the INTERHEART Study which evaluates risk factors for myocardial infarction (MI), or Environment and Global Health Research Group at the Imperial College London, which collaborates with the WHO to provide national–level updates on risk factors such as the prevalence of obesity, hypercholesterolemia, hypertension and hyperglycaemia.

The “big data” approach to global health metrics, currently championed by IHME and increasingly adopted by other groups, should work well over time. The application of sophisticated analytical methods to these massive data sets should be expected to yield population health estimates that would continuously improve over time. However, there are also problems with reliance on “big data” and the field of global health metrics is particularly prone to some of the most frequently highlighted concerns [1]. The much larger amount of data under study will not necessarily make the estimates of disease burden more accurate if most of the data are systematically biased. Moreover, there is a false assumption that very large amount of data automatically implies that the collection will cover all parts of the world adequately and represent underlying populations well. Unfortunately, the global health data available in the public domain today suffers from both these problems. Despite the increasing availability of massive data sets of population–based data the field of global health metrics still faces a number of important challenges.

First, a lot of population health data that is readily available in the public domain, or to organizations such as the WHO or IHME, are national–level estimates based on reporting to national epidemiological services. There is a possibility that these estimates suffer from systematic under–reporting, resulting in estimates that are much lower than the actual situation, even although they are based on very large amounts of data. Second, although the current global health estimates by both WHO and IHME for 2013 make a laudable attempt to model the estimates at the national level for each country in the world, this gives a false impression that there is relevant information available from all these countries. In reality, there are some parts of the world in which there is an abundance of information and the estimates are very precise. However, there are also other regions, and also certain diseases and risk factors, for which the amount of information is remarkably scarce. For some of these countries (or sub–national areas) and conditions the situation has not improved over the past 20–30 years, leading to very large degrees of uncertainty in disease burden estimates. The investment in and development of ever more sophisticated methods of computation and/or epidemiological modeling is less important to achieving valid disease burden estimates – which reflect the true burden in the world population – than investment in generating a sufficient amount of reliable information in those countries and conditions.

These inherent uncertainties are further compounded by the lack of complete transparency from the IHME over their input data that could allow other investigators to replicate their computations and assess the true amount of uncertainty in many of their estimates. In fairness to IHME, though, very few researchers outside the field of global health metrics can truly comprehend the scale of their effort and the size of their data sets, so it is perhaps not surprising that they may feel that they need to continue to refine their data sets and methods through further iterations before they are ready to fully expose them to the rest of the global health research community. [2] However, as long as they do not open their input data and all their methods to a full independent replication by other legitimate academic groups, who do understand these issues – and there are several groups that could do this – it will continue to be difficult for the global health community to fully accept IHME’s estimates. Consistent replication of any scientifically produced result by independent research groups has been a norm in other fields of science, making the key difference between an initial report and a broadly accepted new knowledge. In the end, IHME should benefit more than any other parties from opening their work to other groups – from getting an independent review and feedback on their work, to gaining scientific legitimacy and obtaining suggestions on where to focus further efforts to continue improving their estimates.

In recent months, the WHO and IHME have started to work together with a group of independent academic researchers from this field and senior staff from the *Lancet* and *PLoS Medicine* journals to improve the reporting of population health estimates and, through this, improve the accuracy and transparency of estimates. The new guidelines – likely to be named GATHER (which would stand as an acronym for the “Guidelines for Accurate and Transparent Health Estimates Reporting”) – should improve practices of both those who generate and report primary information on health estimates and those who assemble the primary information and model it to develop global, regional and national health estimates for diseases and risk factors. Once adopted fully, the new reporting guidelines will represent a very helpful step forward that should benefit the field. Successful collaboration of the WHO and IHME teams and international academic experts (including from our Centre for Global Health Research in Edinburgh) on finalizing these guidelines would be a very welcome development, especially if adherence to such guidelines becomes a requirement for publication in all the leading medical journals [3].

We may conclude that, at this point, many positive developments are occurring in the field of global health metrics. The introduction of “big data” approaches, the development of more sophisticated and improved analytic methods, and improved use of new information technologies for data storage and visualization are all contributing to progress. The introduction of new guidelines should add to this progress and generate more papers with primary health data that would be of sufficient reporting quality to be useful for inclusion in different epidemiological models. They will also help to clarify which input data are being used in the models and how the models work.

However, as noted above, there is also a need to increasingly focus on how to generate a lot more information on disease burden and risk factors from “gap” areas of the world. This is particularly true for diseases and risks on which there is hardly any data or epidemiological research in recent decades, and where none of these elements of progress will be able to lead to trustworthy estimates. Possible approaches to address these gaps will need to include research capacity building in gap countries and regions in conducting and reporting epidemiological research. A network of international medical journals interested in global health – such as our *Journal of Global Health* – could play a substantial role in this capacity building. There is a need to educate both the researchers and the journal editors in less developed regions of the good practices and adherence to new guidelines in reporting their health estimates. It is possible that new technologies – such as mHealth and eHealth, ie, the use of mobile phones and internet to gather information – may also enable forms of “crowd–sourcing” approaches to generate population health data in the areas of the world where no other approaches can guarantee success and to understand burden of health problems in real time.

In recent years, our Edinburgh–based group has “specialized” in finding useful health information and developing population health estimates for epidemiologically under–researched problems and areas [4]. This led to the award of the status of the World Health Organization’s Collaborating Centre for Population Research and Training. Our “gap–filling” efforts include trying to learn more about the emerging and alternative sources of medical literature and health information, such as recently digitalized Chinese medical databases CNKI, WanFang, VIP and others. This led to much improved estimates for several major health issues in transitioning China, such as a dramatic reduction of child mortality and its likely causes [5,6], a much finer resolution of the causes of mortality from childhood accidents [7], or the first comprehensive estimates of the burden of dementia [8] and schizophrenia [9] among the adult and elderly Chinese population. For the African continent, we have provided estimates of dementia, COPD, epilepsy, colorectal cancer and rheumatoid arthritis. Likewise, for South Asia, we have published estimates for type 2 diabetes [10–14]. In this current journal issue, we are also publishing a study that estimated of the burden of rheumatoid arthritis in LMICs that was largely based on information from non–English databases and the so–called ‘grey literature’ [15]. We used similar approaches to develop global, regional and, national (wherever allowed by data) estimates for rather neglected and under–researched problems in global health such as childhood pneumonia [16,17], peripheral arterial disease [18] and sequelae from childhood meningitis [19].

Perhaps even more relevant to this “gap–filling” agenda, our group in Edinburgh also pioneered the approach of “academic crowd–sourcing” to address some health issues of specific interest, for which remarkably few data are available in the public domain. As an example, in our attempts to estimate the global, regional and national burden of RSV and influenza infections in children – both of which are important because of a possible opportunities for immunization – we gathered a group of well–minded independent experts who were in possession of either published or unpublished useful information on these under–researched topic, and who agreed to share these data for the purposes of developing global, regional and national estimates [20–22]. Through this approach, we have found out that there is much more useful information available than could be concluded based on reviews of published sources. However, many of the most useful data sets were from studies established for other purposes such as data from control arms of randomized controlled trials which have a disease of interest as an outcome, or data from surveillance systems such as that set up to monitor the evolution of influenza and act as an alert mechanism for viruses with pandemic potential. Mobilizing these valuable sources of data have greatly improved that information available to burden of disease models compared to what was available solely through publically available sources [20–22].

This is precisely where we would like to position a new initiative – the “*Global Health Epidemiology Reference Group*” (GHERG). We would like to propose an informal and entirely voluntary international collaboration of academic groups willing to contribute to improving disease burden estimates to complement IHME activities and who agree to respect the principles of the new guidelines – a form of “academic crowd–sourcing”. Most importantly, all the input data, methods and work should be fully transparent and accessible to all other qualified researchers to verify and replicate them. Ideally, all GHERG papers should have more than one research group involved, and all of the collaborating groups would need to have full access to data. GHERG should, therefore, become an extension of Child Health Epidemiology Reference Group (CHERG), aiming to address global health issues in age groups beyond 0–4 years.

The overall goal of GHERG will be to develop and deploy new and improved evidence on the causes and determinants of morbidity and mortality among populations in all world regions, on the importance of a broad range of risk factors, and on the effectiveness of public health interventions, to inform and influence global priorities and programs. The main focus will continue to be on identifying the “gap areas” where not much information is available and where there is a lot of uncertainty present about the accuracy of the existing estimates. This approach should serve to complement the existing WHO and IHME estimates and to represent added value to both efforts.

The main purposes of the GHERG will be to publish papers, reports and reviews on global health epidemiology, with a special focus on identifying information of sufficient quality in low and middle–income countries and filling the gaps in information for regions where the data are very scarce and of insufficient quality. Also, we would like to advise WHO and other international organizations, institutions and initiatives on the most appropriate methods and assumptions for their global, regional and country level epidemiological estimates. We will aim to advise researchers and public health officials on the different issues involved in the estimation of cause–specific morbidity and mortality.

The core membership of GHERG will initially be offered to the editors and regional editors of the *Journal of Global Health*, who are all independent researchers working for the leading academic institutions. However, GHERG will be open to literally everyone to contribute their data, methods and estimates. It will aim to serve global health community by providing unrestricted open access to its data sets, methods and publications, and continuously revising and updating global health estimates for a targeted set of conditions.

Accurate global health estimates are extremely important because they expose the key issues, inform health policies, direct funding disbursements and eventually solve problems and save lives. In that sense, they could be viewed as a “matter of life and death”. Because of this, when it comes to global health estimates, we see value in both collaboration and competition. Two, three or even more estimates of the same problem, generated by independent research groups, are certainly more informative and helpful than only one – especially if all of them are fully transparent and their methods can be compared [2]. If the principles of the new guidelines are respected by all research groups in the field, then all different estimates would eventually be expected to converge to the same, reliable set of estimates that we are hoping to deliver to the global health community for their unrestricted use.

## References

[1] Marcus G, Davis E. Eight (no, nine!) problems with big data. The New York Times. 6 Apr 2014. Available: http://www.nytimes.com/2014/04/07/opinion/eight-no-nine-problems-with-big-data.html?_r=0. Accessed: 5 Jun 2015.

[2] Rudan I, Chan KY (2015). Global health metrics needs collaboration and competition.. Lancet.

[3] Pan European Networks. WHO and IHME sign MoU. Available: http://www.paneuropeannetworks.com/health/who-and-ihme-sign-mou/. Accessed: 5 Jun 2015.

[4] Rudan I, Lawn J, Cousens S, Rowe AK, Boschi-Pinto C, Tomaskovic L (2005). Gaps in policy-relevant information on burden of disease in children: A systematic review.. Lancet.

[5] Rudan I, Chan KY, Zhang JS, Theodoratou E, Feng XL, Salomon JA, WHO/UNICEF's Child Health Epidemiology Reference Group (CHERG) (2010). Causes of deaths in children younger than 5 years in China in 2008.. Lancet.

[6] Feng XL, Theodoratou E, Liu L, Chan KY, Hipgrave D, Scherpbier R (2012). Social, economic, political and health system and program determinants of child mortality reduction in China between 1990 and 2006: A systematic analysis.. J Glob Health.

[7] Chan KY, Yu X-W, Lu J-P, Demaio AR, Bowman K, Theodoratou E (2015). Causes of accidental childhood deaths in China in 2010: A systematic review and analysis.. J Glob Health.

[8] Chan KY, Wang W, Wu JJ, Liu L, Theodoratou E, Car J, Global Health Epidemiology Reference Group (GHERG) (2013). Epidemiology of Alzheimer's disease and other forms of dementia in China, 1990-2010: a systematic review and analysis.. Lancet.

[9] Chan KY, Zhao FF, Meng S, Demaio AR, Reed C, Theodoratou E (2015). Urbanization and the prevalence of schizophrenia in China between 1990 and 2010.. World Psychiatry.

[10] George-Carey R, Adeloye D, Chan KY, Paul A, Kolčić I, Campbell H (2012). An estimate of the prevalence of dementia in Africa: A systematic analysis.. J Glob Health.

[11] Adeloye D, Basquill C, Papana A, Chan KY, Rudan I, Campbell H (2015). An estimate of the prevalence of COPD in Africa: a systematic analysis.. COPD.

[12] Paul A, Adeloye D, George-Carey R, Kolčić I, Grant L, Chan KY (2012). An estimate of the prevalence of epilepsy in Sub-Saharan Africa: A systematic analysis.. J Glob Health.

[13] Dowman B, Campbell RM, Zgaga L, Adeloye D, Chan KY (2012). Estimating the burden of rheumatoid arthritis in Africa: A systematic analysis.. J Glob Health.

[14] Cheema A, Adeloye D, Sidhu S, Sridhar D, Chan KY (2014). Urbanization and prevalence of type 2 diabetes in Southern Asia: A systematic analysis.. J Glob Health.

[15] Rudan I, Sidhu S, Papana A, Meng SJ, Xin-Wei Y, Wang W, Global Health Epidemiology Reference Group (GHERG) (2015). Prevalence of rheumatoid arthritis in low- and middle-income countries: A systematic review and analysis.. J Glob Health.

[16] Walker CL, Rudan I, Liu L, Nair H, Theodoratou E, Bhutta ZA (2013). Global burden of childhood pneumonia and diarrhoea.. Lancet.

[17] Rudan I, O'Brien KL, Nair H, Liu L, Theodoratou E, Qazi S, Child Health Epidemiology Reference Group (CHERG) (2013). Epidemiology and etiology of childhood pneumonia in 2010: estimates of incidence, severe morbidity, mortality, underlying risk factors and causative pathogens for 192 countries.. J Glob Health.

[18] Fowkes FG, Rudan D, Rudan I, Aboyans V, Denenberg JO, McDermott MM (2013). Comparison of global estimates of prevalence and risk factors for peripheral artery disease in 2000 and 2010: a systematic review and analysis.. Lancet.

[19] Edmond K, Clark A, Korczak VS, Sanderson C, Griffiths UK, Rudan I (2010). Global and regional risk of disabling sequelae from bacterial meningitis: a systematic review and meta-analysis.. Lancet Infect Dis.

[20] Nair H, Nokes DJ, Gessner BD, Dherani M, Madhi SA, Singleton RJ (2010). Global burden of acute lower respiratory infections due to respiratory syncytial virus in young children: a systematic review and meta-analysis.. Lancet.

[21] Nair H, Brooks WA, Katz M, Roca A, Berkley JA, Madhi SA (2011). Global burden of respiratory infections due to seasonal influenza in young children: a systematic review and meta-analysis.. Lancet.

[22] Nair H, Simőes EA, Rudan I, Gessner BD, Azziz-Baumgartner E, Zhang JS, Severe Acute Lower Respiratory Infections Working Group (2013). Global and regional burden of hospital admissions for severe acute lower respiratory infections in young children in 2010: a systematic analysis.. Lancet.

